# Ex-Utero Intrapartum Treatment (EXIT): indications and outcome in fetal cervical and oropharyngeal masses

**DOI:** 10.1186/s12884-020-03304-0

**Published:** 2020-10-07

**Authors:** Lutgardo García-Díaz, Angel Chimenea, Juan Carlos de Agustín, Antonio Pavón, Guillermo Antiñolo

**Affiliations:** 1 Department of Materno-Fetal Medicine, Genetics, and Reproduction, Institute of Biomedicine of Seville (IBIS), Hospital Universitario Virgen del Rocio/CSIC/University of Seville, Avda. Manuel Siurot s/n ES–41013, Seville, Spain; 2 Fetal, IVF and Reproduction Simulation Training Centre (FIRST), Seville, Spain; 3 Department of Pediatric Surgery, Hospital Universitario Gregorio Marañón, Madrid, Spain; 4 Department of Neonatology, Hospital Universitario Virgen del Rocio, Seville, Spain; 5 Centre for Biomedical Network Research on Rare Diseases (CIBERER), Seville, Spain

**Keywords:** Fetal surgery, *Ex-Utero* Intrapartum treatment (EXIT), Placental support, Fetal airway, Airway management, Neck mass

## Abstract

**Background:**

The “Ex-Utero Intrapartum Treatment” (EXIT) procedure allows to ensure fetal airway before completion of delivery and umbilical cord clamping while keeping uteroplacental circulation. Airway obstruction in fetal oropharyngeal and cervical masses can be life-threatening at birth. In those situations, controlled access to fetal airway performed by a trained multidisciplinary team allows safe airway management, while feto-maternal circulation is preserved. We aim to review the indications and outcome of the EXIT procedure in a case series of fetal cervical and oropharyngeal masses.

**Methods:**

We have carried out a retrospective review of all patients with fetal cervical and oropharyngeal masses who underwent an EXIT procedure between 2008 and 2019. Variables evaluated included indication for EXIT, ultrasound and MRI findings, the need of amnioreduction, gestational age at EXIT, birth weight, complications, operative time, survival rate, pathological findings, and postnatal evolution. Five patients are included in this series. One additional case has already been published.

**Results:**

The diagnosis were cervical teratoma (*n* = 1), epulis (*n* = 1) and lymphangioma (*n* = 3). Polyhydramnios was present in 2 patients, requiring amnioreduction in one of them. Mean gestational age at EXIT was 36–37 weeks (range, 34–38 weeks). Median EXIT time in placental support was 9 min (range, 3–22 min). Access to airway was successfully established in EXIT in all cases. All children born by EXIT are currently healthy and without complications.

**Conclusion:**

The localization and characteristics of the mass, its relationship to the airway, and the presence of polyhydramnios seem to be major factors determining indications for EXIT and clinical outcome.

## Background

The “Ex-Utero Intrapartum Treatment” (EXIT) procedure allows ensuring fetal airway before completion of delivery and umbilical cord clamping while keeping uteroplacental circulation. Although EXIT was initially designed to reverse tracheal occlusion performed on fetuses with a severe congenital diaphragmatic hernia, its indications have expanded over the years [1–3].

Airway obstruction in fetal oropharyngeal and cervical masses can be life-threatening at birth. Nowadays, prenatal diagnosis of fetal anomalies allows anticipating an emergent situation, with high fetal morbidity and mortality at birth, by planning the end of the pregnancy. In those situations, controlled access to fetal airway performed by a trained multidisciplinary team allows safe airway management, while feto-maternal circulation is preserved.

The aim of this report is to present our experience with EXIT procedure for the management of fetuses with those pathologies, as well as to review clinical criteria to indicate an EXIT procedure in fetal cervical and oropharyngeal masses.

## Methods

We have carried out a retrospective review of the EXIT procedures related to cervical or oral tumors performed at our Department between January 2008 and December 2019 (*n* = 5, in addition to a case previously published by our team) [4]. Our EXIT surgical approach is described in Table 1. Clinical data and fetal as well as maternal outcomes are summarized in Table 2.

**Table 1:** Summary of the EXIT surgical technique in our Department

EXIT DESCRIPTION	
**1. Personnel:** Multidisciplinary team including anesthesiologists, pediatric surgeons, neonatologists, maternal-fetal medicine specialists, and operating room nurses.	
**2. Maternal anesthesia:** Deep general anesthesia is used, in addition, an epidural catheter is placed to facilitate postoperative pain management of the mother. General anesthesia induction (remifentanil, propofol and rocuronium) is followed in rapid sequence by intubation and assisted ventilation. Before the uterine incision, deep inhalational anesthesia with sevoflurane is used to maintain uterine relaxation and preserve uteroplacental circulation and fetal gas exchange.	
**3. Access to the uterine cavity:**	
a. Low transverse laparotomy.	
b. Once the uterus is exposed, intraoperative sterile ultrasonography is used to	
c. map, carefully, the position of placenta and fetus.	
d. The location of the hysterotomy is determined by the placental locations, and a margin of at least 5 cm from the lower placental edge is left.	
e. Uterine progressive distractor, Satinsky vascular clamps, and a stapling device (Premium Poly Cs-57 Autosuture®) are used in this order to enter into the amniotic sac with minimum uterine bleeding (Fig. 1).	
f. Amnioinfusion with Rintgen’s solution is performed to keep uterine volume.	
**4. Fetal exposure:** A gentle fetal extraction with the help of a single-use suction vacuum (Kiwi©) is performed and the fetus is exposed to the shoulders.	
**5. Fetal airway management:** Fetal anesthesia is supplemented by an intramuscular shot (fentanyl, vecuronium, and atropine) immediately after fetal exposure. Then, the fetal head is positioned to allow access to the airway by direct laryngoscopy or bronchoscopy.	
**6. Delivery:** Once the fetal airway management is completed and secured, the umbilical cord is clamped and divided. The placenta is delivered, the uterine tone is restored (carbetocin plus oxytocin). Finally, uterus and maternal abdominal wall are closed similar to a cesarean section.

**Table 2:** Summary of fetal and maternal outcomes

Case	Single /twin pregnancy	Maternal age (years)	GA at diagnosis (weeks)	Ultrasonographic findings	Prenatal MRI	Preoperative interventions	GA at birth (weeks)	Duration of EXIT (min)	Neonatal procedure	Fetal weight (g)	Airway obstruction	Postnatal therapy	Pathological diagnosis
1	Single pregnancy	28	30	Cystic left-cervical mass size of 65 × 50 × 50 mm, extending from parapharyngeal space at cavum level to supraclavicular space	Yes	No	38	6	Endotracheal intubation	3407	++	Surgical resection	Teratoma
2	Single pregnancy	37	34	Pediculated solid oropharyngeal mass with a maximum diameter of 40 mm	Yes	No	37	3	Endotracheal intubation by bronchoscopy	3100	+	Surgical resection	Epulis
3	Single pregnancy	30	20	Cystic right-cervical mass with a maximum diameter of 63 mm. The tumor crosses the midline and enters the upper mediastinum, compressing and displacing pharynx and larynx. Severe polyhydramnios	Yes	No	36	22	Endotracheal intubation	2900	++	Surgical resection and sclerosing substance injection	Lymphangiomatosis
4	Single pregnancy	28	36	Large macrocystic cervical mass with a maximum diameter of 80 mm. The tumor enters the upper mediastinum, with a tracheal and esophageal displacement	Yes	No	37	7	Endotracheal intubation	3170	++	Treatment with sirolimus	Not performed (mass not excised)
5	Single pregnancy	41	34	Cystic bilateral -cervical mass size of 60 × 56 × 33 mm, with deep facial infiltration and polyhydramnios	Yes	No	36	9	Endotracheal intubation	2889	+	Treatment with sirolimus	Not performed (mass not excised)

### Case 1

A 28-year-old patient, gravida 1, was referred to our center at 37 weeks for evaluation of a cervical mass. 2-D, 3-D, and 4-D ultrasound revealed a homogeneous mass of 6.5 × 5 × 5 cm in the fetal neck, suggestive of a cervical teratoma with tracheal displacement. Amniotic fluid was normal.

The patient underwent an EXIT procedure at 38 weeks’ gestation. The airway was secured by conventional intubation with an endotracheal tube. Time in placental support was 6 min. The newborn weighed 3407 g.

At 12 h of life, the patient underwent a left cervico-lateral mass resection and left hemithyroidectomy without complications, but required mechanical ventilation 48 h following intervention due to marked inspiratory stridor consistent with laryngotracheomalacia. Histopathological diagnosis was immature benign teratoma. Mother was discharged on the fourth day after intervention. Annual monitoring is being performed without evidence of relapsed pathology. The child is currently healthy and without complications at the age of 11.

### Case 2

A 37-year-old patient, gravida 2, para 1, was referred to our center for evaluation of an oropharyngeal fetal mass at 34 weeks. 2-D, 3-D, and 4-D ultrasound revealed a lobulated, solid tumor of 3,7 × 3,6 mm with a thick pedicle dependent on the upper jaw and left nostrils not affecting the fetal neck. Amniotic fluid was normal.

The patient underwent an EXIT procedure at 37 weeks. Fetal airway was secured using a flexible laryngo-fiberscope. Time in placental support was 3 min.

The newborn weighed 3100 g. In the following hours the tumor, pedicled to the premaxilla gingiva, was resected. Histopathological diagnosis was congenital granular epulis.

Mother was discharged on the fourth day after intervention. The child is currently healthy and without complications at the age of 5.

### Case 3

A 30-year-old patient, gravida 1 was referred to our hospital at 33 weeks for evaluation of a facial and cervical fetal tumor. 2-D, 3-D, and 4-D ultrasound showed a giant cervical lymphangioma. MRI confirmed the diagnosis of a large lymphangioma with tracheal and esophageal compression and displacement. Mild polyhydramnios was present.

At 36 weeks the patient underwent an EXIT procedure. Fetal airway was secured by conventional intubation with an endotracheal tube. Time in placental support was 22 min. The newborn weighed 2900 g. On the fifth day of life tumor was partially resected. Injection of sclerosing substances (doxycycline and OK432) was subsequently performed, as well as medical treatment with sildenafil to complete therapy. Despite treatment, at 3 months of age, a respiratory failure developed, requiring a tracheostomy, which was eventually closed at 18 months of age. The mother was discharged on the fourth day after intervention. The child is currently healthy and without complications at the age of 3.

### Case 4

A 28-year-old patient, gravida 1, was referred at 36 + 4 weeks for evaluation of a large cervical lymphangioma. By 2-D, 3-D, and 4-D ultrasound a large and macrocystic cervical lymphangioma, with a maximum diameter of 8 cm, was observed. The MRI confirmed the lymphangioma extending up to the mediastinum, as well as tracheal and esophageal displacement. Amniotic fluid was normal.

Exit surgery was performed in week 37 + 6 days. The airway was secured using a flexible laryngo-fiberscope. Time in placental support was 7 min. Newborn weight was 3170 g. The neonate started treatment with sirolimus (0.04 mg/m2 /day) on the 10th day of life and was successfully extubated at 11 days of life. In this case, no surgical resection was performed.

Mother was discharged on the fourth day after intervention. The patient is currently 6 months old and is being followed by the Pediatric Surgery Department. The child has not needed additional therapy, and his evolution is being good.

### Case 5

A 41-year-old patient, gravida 3 para 2, was referred to our department at 34 weeks for evaluation of a large bilateral cervical cystic lymphangioma with polyhydramnios. Ultrasound scan confirmed a large and macrocystic lymphangioma in the face and the neck. MRI showed a complex cystic lymphangioma with deep facial infiltration and polyhydramnios.

After lung maturation with betamethasone, EXIT was performed in week 36. Airway was secured with flexible laryngo-fiberscope. Time in placental support was 9 min. Newborn weight was 2889 g. The neonate was successfully extubated on the 7th day of life, and treatment with sirolimus was started from the 8th day of life. In this case, no surgical resection was performed.

Mother was discharged on the fourth day after intervention. The newborn was discharged at 22 days of life. The patient is currently 6 months and is being followed by the Pediatric Surgery Department. The boy has not needed additional therapy, and his evolution is being good.

## Discussion

2D, 3D, 4D ultrasound technologies are currently the basis for the diagnosis of different fetal pathologies, including cervical and oropharyngeal tumors as described in our series and other reported cases [5]. In addition, fetal-MRI with a specific sequence can help to improve the information provided by fetal ultrasound [6].

Fetal cervical and oropharyngeal tumors represent very rare entities, whose major problems may be related to the invasion of facial, pulmonary, or central nervous system structures, with the consequent development of polyhydramnios and fetal hydrops. Also, newborns may present an airway obstruction and/or injury due to an intrinsic lesion in the larynx or trachea, or related to an external compression that oropharyngeal or neck masses may produce [2, 4, 7–10]. For that reason, the EXIT procedure has become a standard of care in those cases and may determine the outcome and survival of newborns with different pathologies affecting fetal airway [11–16]. Thus, an increasing trend to the use of EXIT surgery to secure the airway in fetuses with potentially life-threatening upper airway obstruction can be observed.

**Fig. 1:**
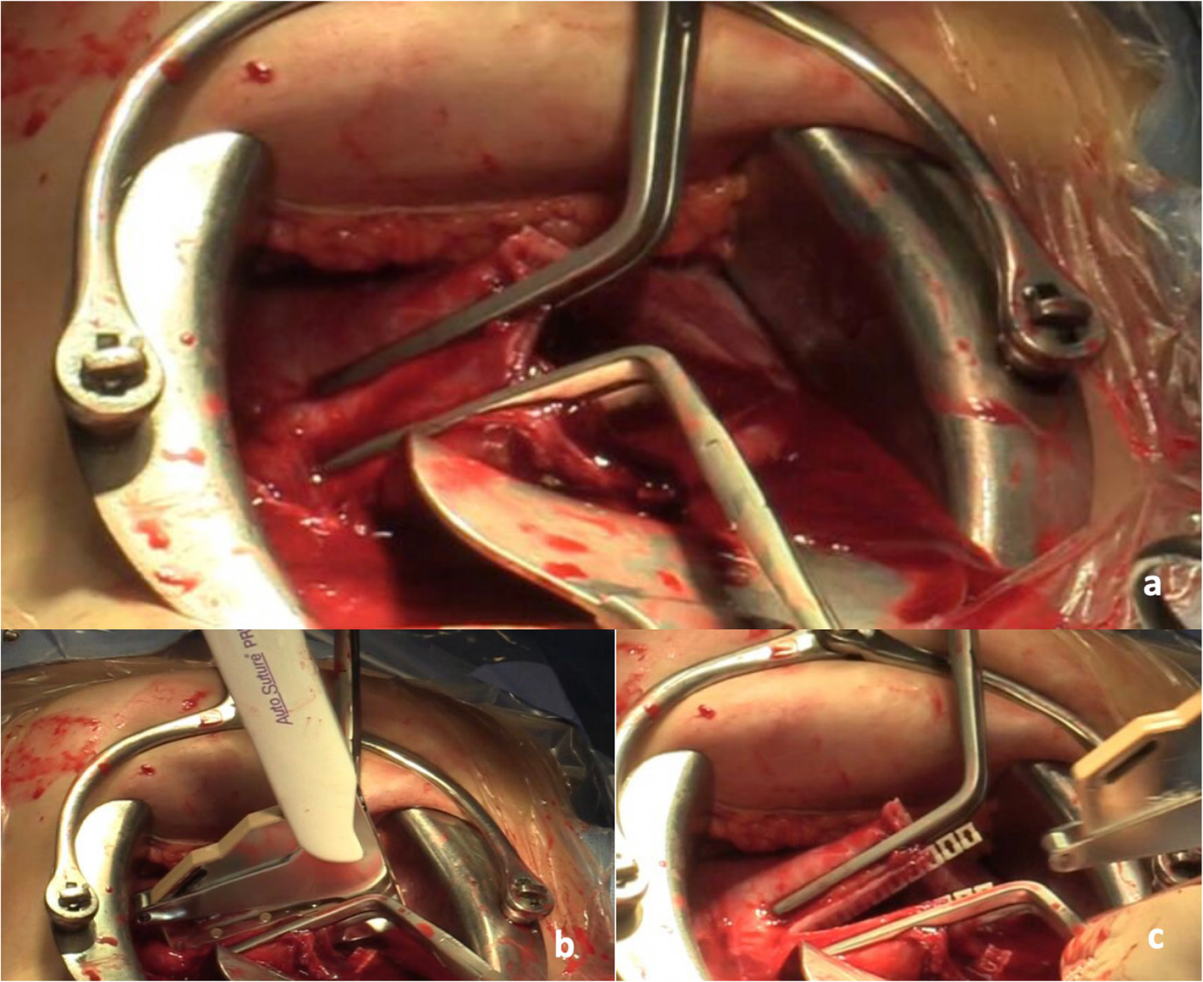
Access to the uterine cavity and amniotic sac: After a low transverse laparotomy and once the uterus is exposed, intraoperative sterile ultrasonography is used to map the position of the placenta and the fetus. Then the access to the uterine cavity and the amniotic sac is made using our atraumatic Uterine Progressive Distractor (**a**), followed by Satinsky vascular clamps (**b**) and a stapling device (Premium Poly Cs-57 Autosuture®) (**c**) to minimize uterine bleeding to allow a safe maternal exit time

Due to potential maternal and fetal risks a careful assessment is necessary before EXIT surgery indication [5, 9, 17–20]. When approached properly by a trained team, EXIT is a safe procedure for mother and fetus. In our surgical technique, summarized in Table 1, a major issue is to secure atraumatic access to the uterine cavity and amniotic sac (Fig. 1) to allow a safe and stable maternal clinical condition during EXIT time.

Novoa et al. [21] recently published a systematic review of the literature evaluating the results of the EXIT surgery in neonatal management of fetal upper airway obstruction. The authors report a fetal or neonatal death rate of 17% (40/235), with an overall fetal adverse event rate of 29.2%. The overall rate of maternal adverse events was 9.4%. These data contrast with those presented in our series of cases, as in our previously published case [4], in which no adverse fetal event occurred, requiring blood transfusion exclusively in one of the cases. The time until maternal discharge was 4 days, similar to that found in a conventional cesarean section in our Department.

## Conclusions

In our experience and others [2, 4, 17–20], in fetuses with cervical and oropharyngeal masses, the localization and characteristics of the mass, its relationship to the airway and the presence of polyhydramnios seems to be major factors determining EXIT approach and clinical outcome. In our series, the presence of polyhydramnios, which may be related to premature labor and/or a neonatal poorer prognosis [6], was consistent with the existence of severe extrinsic obstruction of fetal airway.

When EXIT surgery is performed by a multidisciplinary trained team using a meticulous atraumatic access to the uterine cavity, the perinatal outcome is greatly improved, being a safe technique for mother and fetus.

## Data Availability

The datasets used during the current study are available from the corresponding author on reasonable request.

## Abbreviations

EXIT: Ex-Utero Intrapartum Treatment
MRI: Magnetic Resonance Imaging
